# Non-Physical and Ambient Sexual Harassment of Women Undergraduate University Students in Canada: A Diary Study

**DOI:** 10.1177/10778012231153369

**Published:** 2023-01-30

**Authors:** Katelin Albert, Amanda Couture-Carron, Erik Schneiderhan

**Affiliations:** 1 Department of Sociology, 8205 University of Victoria, Victoria, BC, Canada; 2 3536 Rowan University, Glassboro, USA; 3 71637 University of Toronto Mississauga, Mississauga, Canada

**Keywords:** sexual harassment, university students, diary study, violence against women, mixed methods

## Abstract

Using a 60-day daily e-diary tool, 117 women undergraduate students reported sexual harassment on a Canadian university campus (4,283 diary surveys, collectively). Participants reported 181 incidents of both ambient sexual harassment (*witnessing* 40 incidents*, hearing* 106 unwelcomed sexual jokes/remarks) and targeted personal *experiences* of non-physical sexual harassment (35 incidents). Qualitative data document students’ descriptions of these encounters and contextualize how these are part of everyday student life. Findings show that students experience this harassment almost daily—in an ongoing, persistent, and normalized way—and that university can be a hostile environment where the possibility of daily unwanted sexual experiences is a lived, endemic reality.

## Introduction

Sexual violence—namely, acts of a sexual nature without a victim's consent,^1^ including non-contact forms, such as sexual harassment (Basile et al., 2014)—is a longstanding and significant problem across college and university campuses in North America (Buchwald et al., 1993; Cantor et al., 2020). Defined as “unwanted sexual advances, requests for sexual favors, and other verbal or physical harassment of a sexual nature” (U.S. Equal Employment Opportunity Commission, n.d.), sexual harassment is a common experience on campus; between one-fifth and three-quarters of women have experienced sexual harassment on campus (Cortina et al., 1998; Kelley & Parsons, 2000; Lorenz et al., 2019). These numbers, however, likely underestimate the problem given that the normalization of everyday forms of direct and indirect sexual violence contributes to their misrecognition and underreporting. Indeed, only one-quarter of students who reported various forms of sexual harassment identified it as such (Cortina et al., 1998). Although direct physical sexual violence such as rape and sexual assault^2^ is readily thought of when we consider this problem, non-physical sexual harassment is one of several types of sexual violence that women encounter in their lives as students. Indeed, a continuum of violence exists which connects the normalized everyday sexual violence (i.e., non-physical forms of violence, such as cat calls) to other forms of physical violence (e.g., sexual assault and rape) (Kelly, 1987; Osborne, 1995), illuminating the pervasiveness of various types of violence that women frequently encounter (Osborne, 1995, p. 638).

In addition to these direct and targeted forms of sexual harassment and violence, many scholars of gendered, sexualized, and racialized violence (Ferris et al., 1994; Gutierres et al., 1994; Noh et al., 2007) reveal that people experience violence indirectly as well. For example, *ambient sexual harassment* (wherein people watch, hear, or know another person is experiencing sexual harassment or physical violence) is a form of violence that can have negative effects similar to those experienced by a direct victim (Glomb et al., 1997). In the everyday lives of students, these direct (explicit, subtle, or normalized) and indirect everyday ambient exposures to sexual violence are pervasive and an everyday part of student culture for students who identify as women (hereafter, women).

Inspired by Kelly's (1987) broad conceptualization of violence, our research (using a 60-day daily e-diary method) reveals the everyday, normalized, and insidious nature of non-physical and indirect sexual harassment in the daily lives of students (both on and off campus)—two forms of sexual violence that are often taken for granted and difficult to view. As part of a larger project on campus-based sexual violence in general, we focus on these forms and specifically ask, what is the nature of both non-physical and ambient sexual violence (which includes witnessing another person experience physical sexual violence) in women students’ daily lives? What occurs, where, and how often? Although we recognize that other persons such as men, transgender people, nonbinary folks, and members of the LGBTQIA2 + community experience sexual violence, our focus in this study is on women (which refers to anyone who self-identifies as a woman) as experiencing sexual violence is a pervasive problem among women (Banyard et al., 2007).

Our findings reveal the unrelenting and (almost) everyday nature of sexual violence for women students. By revealing the normalized nature of non-physical and ambient sexual harassment, our paper not only calls for broadening the conceptualization of sexual violence to encompass non-physical and ambient forms, but also suggests that these types of violence must be meaningfully reflected upon and taken seriously within colleges and universities. In addition to broadening the conceptual and practical scope, we argue that all incidents of sexual violence must be contextualized and understood as everyday occurrences in students’ lives (both on and off campus), wherein they are often normalized as a part of students’ experiences. Only then can we improve student life, mental health, and campus culture.

## Sexual Harassment, Campus, and Student Life

Most post-secondary institutions in Canada have worked to establish policies and procedures to address campus-based sexual harassment and many now draw attention to topics like consent, acquaintance rape, rape culture, rape myths, and a culture that discourages reporting and prosecuting cases of sexual violence (Hall & Sandler, 1984; Koss et al., 1985; Warshaw, 1988; Young, 1992). Universities have developed and implemented many programs and policies in response to the problem (see Quinlan et al., 2016). Notwithstanding individual universities’ efforts to shift campus culture, incidents of sexual violence on Canadian campuses continue to be a part of the daily news. Effective sustainable change has been elusive for a variety of reasons. For example, in 2016, the Ontario provincial government implemented Bill 132: Sexual Violence and Harassment Action Plan Act (Ontario, 2016) requiring that all government-funded colleges and universities independently develop sexual violence and sexual assault policies by 2017 (Lopes-Baker et al., 2017; Takagi, 2019). Bill 132, however, did not provide clear guidelines on what to include in such policies (CBC, 2017).

Many interventions, prevention campaigns, and programs continue to use language signaling overt “violence” and “assault” and prioritize extreme forms such as rape and rape culture, often neglecting or making invisible the more subtle, insidious, and ambient forms of sexual harassment. As McMahon and Baker (2011) aptly point out, while the necessity of a broad conceptual term that captures a range of harassment and assault may be familiar and welcomed by scholars and advocates, “the community at large retains a much narrower definition of sexual violence, understanding it mostly as vaginal penetration” (p. 3). While academic and advocacy circles may recognize the importance of subtle unwanted experiences, some still view universities’ emphasis on physical or explicit forms of violence as “appropriate at the current moment…[and] to focus their limited resources on less ambiguous and more serious forms of assault and harassment^3^.”

Even with an emphasis on physical forms of violence, cultural norms at universities are shifting and students are becoming more aware of political correctness and rape myths, which means discourses around sexual harassment are changing as well (see Frazier et al., 1994; Gerger et al., 2007; McMahon & Baker, 2011). As culture changes, however, discourse around rape and sexual harassment become *more subtle or covert* than explicit and overt (O’Connor et al., 2018) and non-physical forms of sexual violence on campus remain pervasive. The media has brought renewed attention to the reality that campus cultures still condone and normalize sexual violence by highlighting campus events such as frosh weeks and pro-rape chants shouted at campuses across the country like the University of British Columbia and Saint Mary's University, and the sexualized social media activity among students at Dalhousie (e.g., Dalhousie suspends 13 dentistry students, 2015; Saint Mary's University unveils, 2014; UBC investigates frosh, 2013). Recently, students at Queen's university displayed signs of threats of sexual violence, harassment, and misogyny during student parties (Ottawa Sun, 2021).

Students mental health and well-being also encompass their lives both on and off campus. While many incidents of sexual violence and harassment occur on campus itself, students’ university experiences extend beyond the walls of campus itself. Moreover, students bring their off-campus experiences onto campus and into university spaces, impacting their time at school, their academic performance, and their campus-based interactions. As such, if we want to understand students’ experiences, we must look to campus culture itself, but also beyond.

## Subtle, Insidious, and Ambient Violence

We are guided conceptually by a feminist praxis that strives to contextualize and make visible women's everyday experiences of harassment. In so doing, we aim to deindividualize harassment, showing the pervasive direct and indirect experiences that are part of women's everyday lives as students. Showing the everyday nature of these experiences emphasizes that these are not isolated and individual experiences, but rather are embedded in the larger culture and everyday life that students move through—both on and off campus. Although sexist beliefs are less overtly endorsed, we begin from the premise that subtle, clandestine, covert, ambient, and indirect forms of discrimination are built into cultural and societal norms, which according to Swim and Cohen (1997), “is why subtle sexism goes unnoticed and is hidden—because it is perceived to be customary or normal behaviour” (p. 104). We take this perspective to establish sexual harassment and assault as a cultural problem rather than an individualized one. This matters in the context of sexual harassment policies and practices that individualize harassment and deal with the experience as an isolated act (Osborne, 1992) where interventions and changes are limited to the individual level with individual behavioral changes.

The effects of subtle discrimination can also have long-lasting effects on women's perception of self. Sexual harassment, such as sexually suggestive speech, can lead to self-objectification (Fredrickson & Roberts, 1997) and women who are targets of suggestive speech are often left frightened, humiliated, and offended (Gardner, 1995). This publicly suggestive speech ranges in scope from “polite” comments, to unsavory, vulgar, offensive, and hate comments. When this occurs, many women are blamed for being targets of sexually suggestive speech, are fearful to say anything about experiencing it, and feel uncomfortable being in public because of it.

Hearing such speech is common and much research documents the detrimental impact and consequences of being targets of mistreatment and harassment. For example, Nielsen (2002) finds that 61% of the women in their sample reported that they are targets of sexually suggestive speech in public places everyday/often; Barling (1996) emphasizes that non-physical aggression is linked to negative psychological and psychosomatic outcomes. Similarly, Fitzgerald et al. (1997) argue that sexual harassment triggers health problems, psychological wellbeing, withdrawal, and lowers job satisfaction. Even when a woman is not the direct target, scholars emphasize the saliency of witnessing harassment, violence, and discrimination—vicarious or ambient harassment. For example, Schmader et al. (2012) find that people can experience serious emotional discomfort if they witness but do not act toward discrimination. Miner-Rubino and Cortina (2007), investigating workplace harassment, argue that vicarious harassment leads to lower wellbeing and higher rates or organizational withdrawal for employees. Barling (1996) explains that when others are exposed to hostility, they are *secondary victims*. Glomb et al. (1997) describe this as *ambient sexual* harassment. Regardless of the term, those who observe sexual harassment also experience negative effects and bystander stress (Hitlan et al., 2006), as a secondary, nontargeted victim (Andersson & Pearson, 1999).

While existing research documents the harmful effects of harassment, we know less about the everyday nature of this violence nor the context within which it occurs among women university students. With our diary approach to studying this issue, we offer a more accurate snapshot of harassment's pervasiveness alongside a qualitative understanding of the nature of these events based on an analysis of how women describe these incidents. Exploring various forms of sexual harassment—targeted nonphysical and ambient sexual harassment—we draw attention to these often overlooked, hidden, and de-prioritized form of violence against women. In so doing, this research establishes that subtle, non-physical, and ambient forms of violence against women are pervasive within the everyday lives of students. Research shows that women are more likely to recognize and take action against overt discrimination, compared to subtle forms (Lindsey et al., 2015). The more that normalized, everyday, persistent, subtle, and ambient forms of violence are not the main focus of universities and colleges, these forms of sexual violence persist. Below, we document them on one campus and demonstrate some of their harmful effects using the words of our respondents. We then suggest strategies for positive change.

## Methods

To expand knowledge and understanding on a continuum of violence, we used an online daily 60-day e-diary survey where students report their experiences of sexual harassment each day (from explicit to more subtle forms), thus increasing the likelihood of accurately capturing the everyday nature and range of more subtle, normalized, and ambient sexual harassment that may not be recalled easily in six months’ time. Used seldomly as a method (for example, see Hirsch et al., 2018; Mellins et al., 2017), this unique approach offers several advantages. Through this method, we were able to ask a wide range of questions about experiencing sexual harassment on campus, including the more mundane, normalized, subtle, ambiguous, and forgotten sexual harassment that “may be quickly brushed off as a function of everyday living and survival” (Swim et al., 2003, p. 41).

A diary study is also unique given that the majority of research on sexual violence utilizes either large-scale survey data (e.g., campus climate surveys) or interview-based data where people share their experiences. While both sets of data are extremely useful for capturing broad trends as well as victims’ experiences, they rely on retrospect and recall memory. For example, in large-scale surveys, a research participant may be asked, whether or how many times one experienced various forms of sexual harassment *since enrollment at the university/college* (see Wood et al., 2021 for timeframe example) or *during the current academic year* (e.g., see Banyard et al., 2007 for timeframe example). Not only is it difficult to recall how many occurrences were experienced (resulting in underreporting), but participants may also only recall obvious or explicit occurrences and not necessarily micro-occurrences, such as witnessing or hearing an unwanted sexual joke, look, etc. When using a diary method, however, participants’ recordings of daily experiences produce more real-time data allowing for greater accuracy in ascertaining the frequency of students’ unwanted sexual experiences. Diaries provide a “dramatic reduction of the likelihood of retrospection, achieved by minimizing the amount of time elapsed between an experience and the account of this experience” (Bolger et al., 2003, p. 580). In other words, when participants report at least daily rather than looking back on past experiences over months or even years, there is a reduction of retrospective bias (Reis & Gable, 2000).

Ohly et al. (2010) also point out that a diary design for data collection is ideal for gathering data that “is highly fluctuating and strongly dependent on situational conditions” (p. 79). Asking students to report witnessing or experiencing sexual harassment every day captures students’ immediate reactions to these events. Moreover, they are more likely to report an incident since they have not yet rationalized their experiences, which may make them less likely to see it as harassment and more likely to excuse it. With this, we also get more detailed information about the event—what, where, and how. This provides the context in which the incidents occur alongside the frequency of the incidents. Our diary study, therefore, contributes to existing scholarship by using this methodological approach and offering unique data.

### Recruitment and Sample

After receiving Research Ethics Board approval, we recruited participants using posters hung throughout the university campus and handbills that members of the undergraduate research advisory committee distributed on campus. Both the handbills and the posters sought participants for a “… pilot study on unwanted sexual contact on university campuses,” and included information on the extent and nature of the involvement, eligibility requirements, monetary compensation, study contact information, funding information, and a link for the Qualtrics secure online survey platform, where the survey was hosted.

Our sample includes 117 participants who were undergraduate students and who self-identified as women. These participants completed an initial baseline survey (described below) and at least one diary survey (see Ohly et al., 2010 for more on noncompliance in diary studies). Collectively, these 117 participants completed 4,283 diary surveys over 60 days. Participation rates ranged from days when 104 women participated (day 10) to days when 40 women participated (day 33).

### Procedures

Upon visiting the Qualtrics survey link, participants gave electronic consent to participate and chose a unique ID code (that only they knew) to use for the duration of this research. Since the data collected is longitudinal, a unique identifier code was needed in order to connect individuals’ multiple diary entries as well as their initial baseline survey. Students began with a one-time online baseline survey (approximately 45 min of time) that gathered data on participants’ demographics, social capital, mental health, substance use, prior sexual victimization, and sexual behavior. Upon completing the baseline survey, participants received a new website link directing them to the daily e-diary survey (also Qualtrics). Participants completed a second consent form for this stage of the research, and then were prompted to complete an e-diary entry consisting of multiple choice and short answer questions about some of the participants’ daily experiences and mental health. The daily diary survey took approximately five minutes to complete—fitting the ideal amount of time earlier research established (Reis & Gable, 2000). We asked participants to return to the same diary survey link every 24 h for 60 days (from January until March 2018), including weekends, to fill out the same diary survey everyday (the survey would be empty when refreshed). A 60-day timeframe is sufficiently long to establish the trends and patterns of typical of a semester.

To ensure continued participation, we used a monetary compensation plan (offering up to $80 in total incentives per student). The plan increased compensation over the length of the study (e.g., $0.50 for days 1 through 20; $1.00 for days 21 through 40; $1.50 for days 41 through 60; weekend completions of $1.00 per weekend day). Daily reminder emails were automatically sent to all participants who had not yet filled out their daily survey. If a participant missed a diary entry, they could continue to participate in the following days. If the participant completed the baseline survey and at least one diary survey, their diary entry was included in the data analysis.

### Survey Design and Measures

Previously implemented surveys on sexual assault, health, and wellness developed by a variety of experts in the social sciences, such as the Canadian Community Health Survey (CCHS, 2016), the Massachusetts Institute of Technology (MIT, 2014), the Center for Collegiate Mental Health (CCMH, 2018), and the Center for Epidemiological Studies (CES, 2004) informed the design of our surveys. We modified questions from established surveys as necessary to make them appropriate for the Canadian and specific university context. The daily diary survey included a wide range of questions about sexual activity, unwanted physical sexual contact, substance use, daily sexual harassment, and mental health questions. For this manuscript, however, the focus is on daily sexual harassment. An undergraduate advisory committee of 10 demographically diverse students from multiple areas of study reviewed the baseline survey and the daily diary survey, provided feedback on our questions, and tested the surveys to ensure language and questions were relevant to their experiences and accessible to the diverse local study body.

As previously noted, the baseline survey gathered data on participants’ demographics, social capital, mental health, substance use, prior sexual victimization, and sexual behavior. For example, the demographic questions assessed participant’ age, gender identity, and race. Sexual behavior-related questions included, for instance, “what type of sexual contact have you had with someone else BEFORE becoming a student at (X university)? (select all that apply).” The current study, however, analyzes only the demographic information from the baseline survey. The core data analysis for this study uses measures of non-physical sexual harassment and witnessing sexual assault from the diary surveys, which are specified below.

#### Ambient Sexual Harassment—Witnessing

To assess indirect and ambient sexual harassment, we captured whether participants witnessed sexual harassment in the daily diary survey. We asked, “Have you witnessed someone experience unwanted sexual contact (this could be physical, verbal, emotional, online or email lists, on social media sites)?” If they had, they were prompted to identify where it occurred and what took place, permitting qualitative analysis. If more than one incident occurred in the 24-h period, participants were asked to select all that applied.

#### Hearing a Sexual Joke or Sexual Remark

To determine whether the participants heard a sexual comment or joke, we asked the following question: “Since you last filled out the diary, did you hear someone make a sexual remark or tell a sexual joke that you found insulting or offensive.” If they had, they were asked to identify where it occurred (selecting all that applied, if more than one incident occurred). This could have been hearing a joke directed at them, or directed at another person.

#### Non-Physical Direct Forms of Sexual Harassment

Participants were asked, “Since you last filled out the diary, have you been sexually harassed (this includes unwelcome sexual advances, requests for sexual favors, and other verbal conduct of a sexual nature)?” If they had, they were asked where the incident occurred and to qualitatively describe what happened.

For all of the above measures, the data analysis reports on the *number of times* participants reported particular responses to our questions. We cannot report these as individual incidents because of possible overlap in survey responses for single participants. For example, a participant may have reported witnessing unwanted sexual contact taking the form of penetration and forced oral sex. We are unable to determine with certainty if the participant is describing a single incident with multiple forms of unwanted contact or two incidents with different types of contact. In light of this, we report the number of times particular responses were chosen. It could also be the case that multiple women reported the same incident. However, this concern for duplicate reporting is not an issue analytically since we show the pervasive and widespread impact of sexual harassment. Multiple people reporting the same incident reveals the everyday nature of students and would only indicate that multiple students are experiencing violence.

### Data Analysis

We analyzed the quantitative data using Stata to identify the frequency of participant responses. Descriptive statistics allow for identifying the trends and patterns in participant experiences. The qualitative data and participants’ descriptions of events are limited, but were imported into qualitative analysis software, Dedoose, and coded using open coding which helped identify patterns and themes in descriptions. The descriptions of the events were usually 1–2 sentences long and were coded to understand the normalized and everyday nature of the incident.

## Results

### Participant Characteristics

As noted, 117 students who self-identified as women participated in both the baseline and diary surveys. Participants ranged in age from 17 to 35 with an average age of 20 years. The majority (75%, *n* = 86) were between the ages of 18 and 20 years of age and in the first two years of their university program (42%, *n* = 49 in the first year; 22%, *n* = 26 in the second year). Fewer were in the third year (19%, *n* = 22) and fourth year (14%, *n* = 16). Most also identified as straight/heterosexual (87%, *n* = 102), while the remainder (13%, *n* = 15) identified as lesbian, gay, bisexual, or other. Almost all participants self-identified as women. The majority of women self-identified as South Asian (39%, *n* = 45) or White (28%, *n* = 32). Participants also identified as Arab (5%, *n* = 6), Chinese (4%, *n* = 5), and other (39%, *n* = 45).

### An Everyday Thing: Sexual Harassment

Our respondents experienced a form of non-physical and ambient sexual harassment almost every day. Plotting these incidents on a 60-day calendar (see Table 1) reveals the unrelenting pervasiveness of harassment and shows that from January 22 to March 22 (60 days), there are 181 incidents of some type of non-physical sexual harassment. It could be the case that multiple students are reporting the same event although this is unlikely as the university is large both physically and in terms of enrollment. In any event, we are not concerned about inflation of the data since one event or incident can be experienced by multiple students and have an impact on multiple people. Indeed, at least one student reported an incident of non-physical sexual harassment on 51 of those 60 days—almost every day. In fact, multiple forms of harassment can be seen on most days. While the number of incidents a day ranges, with day one having 23 incidents, between 2 and 5 incidents a day is most common. Several of the days that have no reported incidents are later in the study timeframe and may be a result of participant-response decline as the diary study progressed. However, given this was a small sample of women students, the everyday and common reporting is particularly concerning, and even more daily incidents might have occurred in a larger, representative study. The persistent nature of these incidents shows that a continuum of violence is an everyday experience for women students.

**Table 1.** Frequency of Non-Physical Sexual Harassment on 60-Day Calendar.

Monday	Tuesday	Wednesday	Thursday	Friday	Saturday	Sunday
**January 22^a^** Witness: 5 Remarks: 16 Harass.^b^: 2 **Total incidents: 23** **# of Part.^c^: 71**	**23** Witness: 2 Remarks: 11 Harass.: 0 **Total incidents: 13** **# of Part.: 98**	**24** Witness: 3 Remarks: 5 Harass.: 1 **Total incidents: 9** **# of Part.: 93**	**25** Witness: 3 Remarks: 5 Harass.: 1 **Total incidents: 9** **# of Part.: 95**	**26** Witness: 1 Remarks: 1 Harass.: 1 **Total incidents: 3** **# of Part.: 92**	**27** Witness: 1 Remarks: 4 Harass.: 0 **Total incidents: 5** **# of Part.: 87**	**28** Witness: 0 Remarks: 3 Harass.: 1 **Total incidents: 4** **# of Part.: 83**
**29** Witness: 1 Remarks: 1 Harass.: 0 **Total incidents: 2** **# of Part.: 98**	**30** Witness: 0 Remarks: 2 Harass.: 1 **Total incidents: 3** **# of Part.: 90**	**31** Witness: 1 Remarks: 4 Harass.: 0 **Total incidents: 5** **# of Part.: 104**	**February 1** Witness: 0 Remarks:1 Harass.: 0 **Total incidents: 1** **# of Part.: 80**	**2** Witness: 2 Remarks: 3 Harass.: 2 **Total incidents: 7** **# of Part.: 88**	**3** Witness: 1 Remarks: 4 Harass.: 2 **Total incidents: 7** **# of Part.: 82**	**4** Witness: 0 Remarks: 2 Harass.: 1 **Total incidents: 3** **# of Part.: 74**
**5** Witness: 0 Remarks: 2 Harass.: 1 **Total incidents: 3** **# of Part.: 81**	**6** Witness: 0 Remarks: 2 Harass.: 0 **Total incidents: 2** **# of Part.: 84**	**7** Witness: 0 Remarks: 2 Harass.: 0 **Total incidents: 2** **# of Part.: 83**	**8** Witness: 0 Remarks: 1 Harass.: 0 **Total incidents: 1** **# of Part.: 76**	**9** Witness: 1 Remarks: 3 Harass.: 0 **Total incidents: 4** **# of Part.: 76**	**10** Witness: 2 Remarks: 0 Harass.: 2 **Total incidents: 4** **# of Part.: 67**	**11** Witness: 2 Remarks: 1 Harass.: 2 **Total incidents: 5** **# of Part.: 79**
**12** Witness: 0 Remarks: 2 Harass.: 0 **Total incidents: 2** **# of Part.: 77**	**13** Witness: 0 Remarks: 1 Harass.: 0 **Total incidents: 1** **# of Part.: 71**	**14** Witness: 1 Remarks: 1 Harass.: 0 **Total incidents: 2** **# of Part.: 71**	**15** Witness: 0 Remarks: 0 Harass.: 1 **Total incidents: 1** **# of Part.: 70**	**16** Witness: 1 Remarks: 0 Harass.: 0 **Total incidents: 1** **# of Part.: 65**	**17** Witness: 1 Remarks: 2 Harass.: 3 **Total incidents: 6** **# of Part.: 67**	**18** Witness: 0 Remarks: 1 Harass.: 0 **Total incidents: 1** **# of Part.: 67**
**19** Witness: 2 Remarks: 1 Harass.: 0 **Total incidents: 3** **# of Part.: 64**	**20** Witness: 1 Remarks: 2 Harass.: 2 **Total incidents: 5** **# of Part.: 67**	**21** Witness: 0 Remarks: 1 Harass.: 0 **Total incidents: 1** **# of Part.: 60**	**22** Witness: 1 Remarks: 1 Harass.: 0 **Total incidents: 2** **# of Part.: 67**	**23** Witness: 0 Remarks: 0 Harass.: 1 **Total incidents: 1** **# of Part.: 40**	**24** Witness: 0 Remarks: 0 Harass.: 0 **Total incidents: 0** **# of Part.: 59**	**25** Witness: 0 Remarks: 0 Harass.: 1 **Total incidents: 1** **# of Part.: 70**
**26** Witness: 1 Remarks: 0 Harass.: 3 **Total incidents: 4** **# of Part.: 73**	**27** Witness: 1 Remarks: 1 Harass.: 0 **Total incidents: 2** **# of Part.: 68**	**28** Witness: 1 Remarks: 3 Harass.: 0 **Total incidents: 3** **# of Part.: 71**	**March 1** Witness: 0 Remarks: 0 Harass.: 1 **Total incidents: 1** **# of Part.: 64**	**2** Witness: 0 Remarks: 0 Harass.: 0 **Total incidents: 0** **# of Part.: 62**	**3** Witness: 0 Remarks: 5 Harass.: 2 **Total incidents: 7** **# of Part.: 65**	**4** Witness: 0 Remarks: 1 Harass.: 0 **Total incidents: 1** **# of Part.: 61**
**5** Witness: 0 Remarks: 0 Harass.: 0 **Total incidents: 0** **# of Part.: 69**	**6** Witness: 1 Remarks: 2 Harass.: 1 **Total incidents: 4** **# of Part.: 61**	**7** Witness: 1 Remarks: 2 Harass.: 0 **Total incidents: 3** **# of Part.: 65**	**8** Witness: 0 Remarks: 0 Harass.: 0 **Total incidents: 0** **# of Part.: 58**	**9** Witness: 1 Remarks: 0 Harass.: 0 **Total incidents: 1** **# of Part.: 57**	**10** Witness: 0 Remarks: 0 Harass.: 0 **Total incidents: 0** **# of Part.: 58**	**11** Witness: 0 Remarks: 1 Harass.: 1 **Total incidents: 2** **# of Part.: 63**
**12** Witness: 0 Remarks: 1 Harass.: 1 **Total incidents: 2** **# of Part.: 80**	**13** Witness: 1 Remarks: 1 Harass.: 0 **Total incidents: 2** **# of Part.: 66**	**14** Witness: 0 Remarks: 1 Harass.: 0 **Total incidents: 1** **# of Part.: 59**	**15** Witness: 0 Remarks: 0 Harass.: 0 **Total incidents: 0** **# of Part.: 56**	**16** Witness: 0 Remarks: 0 Harass.: 0 **Total incidents: 0** **# of Part.: 60**	**17** Witness: 0 Remarks: 0 Harass.: 0 **Total incidents: 0** **# of Part.: 55**	**18** Witness: 0 Remarks: 0 Harass.: 0 **Total incidents: 0** **# of Part.: 67**
**19** Witness: 0 Remarks: 1 Harass.: 0 **Total incidents: 1** **# of Part.: 59**	**20** Witness: 0 Remarks: 1 Harass.: 0 **Total incidents: 1** **# of Part.: 79**	**21** Witness: 1 Remarks: 1 Harass.: 1 **Total incidents: 3** **# of Part.: 51**	**22** Witness: 0 Remarks: 0 Harass.: 0 **Total incidents: 0** **# of Part.: 60**			

a Calendar date.

b Harassment.

c Participants.

In what follows, we elaborate on the everyday, normalized, and persistent nature of sexual harassment in students’ lives. We report the frequency, nature, and context of the three types of harassment that are captured in this calendar—(a) *witnessing* sexual harassment, (b) *hearing* offensive sexual comments or jokes, and (c) personally *experiencing* non-physical forms of sexual harassment.

### Witnessing Harassment—Indirect and Ambient Sexual Harassment

Witnessing sexual violence, assault, and harassment is a type of ambient sexual violence that creates indirect secondary victims to direct violence, producing a culture of fear for women students (Kelly & Torres, 2006). Over the 60 days, our data reveal that students reported witnessing harassment 40 times. In many instances, students witnessed harassment in person, while others observed harassment online, and some heard about the harassment from a friend telling them about an experience or showing them something that happened to them. In all of these instances, the unwanted sexual experience was not directed towards the participants themselves, but they witnessed it firsthand or became aware of the incident from the direct target (e.g., from a friend), thereby becoming a witness, so to speak. Kelly and Torres (2006) argue that knowing that a friend has been victimized impacts women students’ fear and perception of safety on campus just as much as one's own experience and personal victimization. Below, we outline these experiences, from witnessing non-physical sexual harassment, hearing a sexual comment or joke, and witnessing physical forms of harassment. To emphasize the normalization of the incidents, we also reveal some qualitative descriptions of the incidents, how they unfolded, including the persistence of the harassment despite resistance from women.

#### Witnessing Non-Physical Sexual Harassment and Hearing a Sexual Comment or Joke

Participants reported witnessing *non-physical* sexual harassment of some kind 26 times, 12 of which they witnessed online. Descriptions of witnessing non-physical incidents can be grouped into three themes: Witnessing someone (a) make sexually explicit comments, (b) use unwelcomed pick-up lines, and (c) make comments about women's bodies.

Qualitative entries include women describing 16 incidents where they witnessed *sexually explicit comments*. For example, one student described witnessing, “a man sent a spew of messages to a girl he matched with on a dating media site saying how he is the king of pussy and other vulgar language to seduce her.” Another student reported witnessing a horrific and disrespectful conversation, hearing someone “[talk] about a girl who was raped and then killed in a somewhat disrespectful manner.” In five descriptions, participants heard comments that can be categorized as *pick-up lines*, or asking for numbers, and are not necessarily sexually explicit, but were deemed as witnessing an unwanted advance (i.e., witnessing sexual harassment). For example, one student reported, “the first incident occurred after class to my roommate where she was approached by someone in her class and was being hit on making her very uncomfortable.” Similarity, another woman said, “someone stopped her, [another student], out of class and was hitting on her and trying to ask for her number [to] go out with him which she kept saying no to, but he wouldn’t stop.” In five other instances, the harassment women witnessed was *directed toward women's bodies*. For example, one student reported, “guys were messaging her, commenting on her body, and when she took offence to it, they got verbally aggressive towards her.” Another said she witnessed “a random person commented, ‘nice tits.’” Referring to an incident that occurred on the bus, one student reported,

A guy followed a girl through the long [local] bus as she changed seats twice to avoid talking to him. He kept commenting on her appearance and her chest, legs, butt and tried to touch her the last time he approached her. Also [asked] her if she was afraid of him. I got up to talk to him and confront him about his unacceptable behaviour when [I noticed] things started to get more serious. No one else seemed [to] want to do anything about it. But before I approached him, the guy left the bus at the next stop, cursing at the girl.

These 26 incidents of witnessing a non-physical harassment reveal a variety of individual experiences but show they occur in many everyday scenarios. They occur in passing and in everyday places such as while taking the bus, leaving class, or being on social media. Witnessing these is an indirect and ambient form of harassment. Hearing a “group of guys” disrespectfully discuss a rape and murder, or male aggression after a witnessing a fellow woman student be offended by a sexual comment creates a toxic environment where women students may not only feel unwelcomed, but also unsafe, particularly when “responding to” or showing resistance to the harassment.

Students were also asked if they had heard someone make a sexual remark or joke that they found insulting or offensive. We include this in witnessing sexual harassment and with this specific question, students reported hearing a sexual remark or joke 106 times. However, students were not asked to qualitatively describe these remarks or jokes. The frequency of hearing a joke or comment further reveals the everyday reality that students face and the normalized ambient harassment that makes up their everyday lives.

#### Witnessing Physical Sexual Harassment

In addition to witnessing non-physical harassment, participants described witnessing incidents that were *physical* in nature 16 times. For example, one student reported seeing a “group of guys fingering her [a girl at a party] while she was drunk;” Another student reported, “a man inappropriately touched one of my friends.” Demonstrating how normalized and persistent witnessing physical sexual assault is, one student listed a string of incidents she saw in one comment: “someone was groped. Someone had their butt grabbed.” Another student witnessed an incident of physical sexual harassment that was filmed and said, “a person [was] filming another getting sexually assaulted and then thanking the other person for not saying anything.”

In some of the descriptions, participants specified that a fellow student told them about physical harassment that occurred. We count these as witnessing the event since they enter into the participant's consciousness, are part of their view, and interpolate the student into the scenario, positioning them as a secondary victim. Moreover, although we did not specifically ask participants to report if a fellow student told them about their experience of harassment, multiple women in this study identified these situations as “witnessing” harassment suggesting they interpreted them as such. For example, one student reported that a fellow “student told [her] that they were intoxicated with a group of guys, and they sexually harassed her in her bedroom.” Another participant described what her friend experienced saying, “someone came onto another [person] and they tried pushing away, but they were quite forceful. Outside help was necessary.” Another woman explained a conversation between her and a friend, saying, “her boyfriend was drunk and kept asking her for sex despite her saying no. She gave in and [told me] it was the worst night of her life. She asked [me] whether it was sexual assault. And I said yes.” Referencing the larger context of student life and consciousness, as well as the everyday macro climate that students are a part, one student just said, “someone was attacked today [at] YorkU.”

The context of witness sexual harassment also involved witnessing its normalized persistence, despite women's resistance, demonstrating the hostile environment in which students exist every day. While we did not specifically ask participants about resistance or resistance responses, many included descriptions of these when reporting what happened. The qualitative descriptions of witnessing reveal the complicated and dynamic ways sexual violence and sexual harassment unfold and give insight into what students are witnessing.

Participants reported that they witnessed resistance in 12 of the 40 incidents of witnessing sexual harassment. In particular, they described 5 cases of verbal resistance (from a woman verbally expressing discomfort to explicit stating “no”), and 5 cases of indirect resistance. Indirect resistance included using body language to convey discomfort and unwantedness and physical avoidance. For example, participants described women physically moving or leaving a space to avoid the harassment: “she was talking to me and a stranger at the event. [He] was eyeing and approaching her trying to talk to her while she was trying to avoid him by talking with me and walking away.” Another woman described an unspecified resistance—“the girl refused to engage in sexual texting and the man told her to kill herself”—where it is unclear if this was verbal or just avoidance. One participant described witnessing physical resistance and said, “they tried pushing away.”

These descriptions of resistance were commonly accompanied by descriptions of rejection responses, where participants described what the male harassers did in response to the resistance. Participants described witnessing these rejection responses 10 times. Some included witnessing men deny that they were sexually harassing the woman. Often, descriptions included persistent and continued harassment even after resistance. Some of the participants described scenarios where the men continued to physically harass the women, others included men continuing to follow the women in spaces or not leave the women alone. Disturbingly, four participants described scenarios where the men became violent, aggressive, and threatening when the women expressed some type of resistance. For example, one woman said, “… when she brushed him off and tried to ignore him, he became hostile and threatened her with more explicit acts.” Similarly, another woman reported, “… when she took offense to it, they got verbally aggressive towards her.” In the two incidents described above, men “cursed her” and “told her to kill herself” after the women expressed resistance.

### Directly Experiencing Non-Physical Sexual Harassment

After asking participants about witnessing sexual harassment and hearing sexual comments or jokes, we asked if they had *personally experienced* any *non-physical* sexual harassment. This includes unwelcome sexual advances, requests for sexual favors, and other verbal conduct of a sexual nature. Over the 60 days, women reported experiencing sexual harassment 35 times. Of the 35 reported incidents of sexual harassment, participants qualitatively described 32 experiences. Notably, while we did ask people about their experiences of physical sexual violence and assault in the daily diary, our analysis for the purposes of this article only focuses on personally experiencing *non-physical* harassment and does not include any personal experiences of unwanted *physical contact* or *sexual assault*.

An analysis of these qualitative descriptions of non-physical harassment reveals that this sexual harassment commonly occurs during everyday events or moments that are not necessarily considered risky. For example, nine participants specifically described sexual harassment during everyday events such as “waiting for a delivery,” “crossing the street,” “someone walking by,” eating in a restaurant, riding the bus, studying in the library, or at a family dinner. The sexual harassment that students reported ranged in nature. The most common types of sexual harassment were catcalling (e.g., “hey sexy,”)—which were often perpetrated by groups of men—and direct and explicit sexual comments or advances. For example, one woman reported that she was in the university library when “a man [an undergraduate student] made a comment about how I should let him put his penis in my mouth.” Other women's descriptions reveal that these comments are made in passing. For instance, one woman said, “someone was commenting on how I looked and was making sexual comments like, ‘I’d tap that’.” Another woman explained, “he said he has had problems ‘getting laid’ and moved closer and closer to me.” Similarly, one student described an incident she experienced on campus:

I fell on the ground and stated, “oh my god I am so wet right now” (I fell in a puddle). He [an undergraduate student] was standing next to me and told me to wait until I saw what he could do.

Other times, students described personally experiencing sexual harassment where they felt they were the sexual subject of someone's gaze. One woman reported, an “old man gave me sexual looks.” Another student said, “I heard an unwanted comment sexualizing my body.” At times, being under someone's gaze involved the use of photos specifically. For example, one woman said, “someone walked by and took a picture of my butt.” Another participant explained, what she experienced was an “invasion of privacy. He took my phone while I was asleep and sent himself provocative pictures of myself from my phone.”

Like above, experiencing non-physical sexual harassment also involved persistent sexual harassment. Qualitative descriptions of personal experiences of non-physical sexual harassment reveal the ongoing nature of these incidents. Eleven descriptions of these incidents included women explaining some form of resistance—verbal, physical avoidance (e.g., crossing the street, moving over), or physically pushing someone away. They describe six instances of verbal resistance (from verbally expressing discomfort to explicitly saying “no”), three instances of an unspecified resistance where it is unclear if it was verbal or not, “just some guys making advances and not taking a hint,” and one instance of indirect resistance, “creepy guy called out to me and yelling louder *when I kept walking*.” One case involved physical resistance, “me pushing them off.”

Similar to witnessing sexual harassment, a few qualitative descriptions of incidents of experiencing sexual harassment conveyed that the perpetrator became agitated, angry, or persistent in response to their resistance or avoidance. While we did not specifically ask about this, participants reported men's reactions to their resistance in their descriptions nine times. Consistent with the findings regarding witnessing harassment, the most common theme in men's reactions to the women's resistance was persistent and continued harassment. While this section reports *non-physical* sexual harassment, these qualitative descriptions reveal the analytic difficulty of separating a sequence of events in sexual harassment. Men's responses to women's resistance commonly reveal how harassment that is initially non-physical (or one that involves both physical and non-physical) might subsequently include some form of physical harassment. For example, six of the student's qualitative descriptions of men's responses to her resistance involved some form of physical harassment and persistence as well. One participant described, “he first talked about my looks and all and said I was fit for him and later asked for my number which I didn’t [give] him. So, he tried to take my phone and grab my hand.” Disturbingly, three participants described scenarios where the men became angry and rude when the women expressed some type of resistance. For example, one woman said, “…he asked if I wanted sex and when he noticed that my boyfriend was beside me he said, ‘well are you in a monogamous relationship?’ When I said yes, he got angry and stormed out of the restaurant.” Another student describes how she “was approached by a guy that made lewd sexual advances. [I] told him I wasn’t interested, and he became annoyed and even more rude before leaving.”

These descriptions of the incidents reveal the persistent nature of harassment, how harassment evolves in a scenario, perhaps ending with a physical assault or escalating harassment. Because of this, large-scale surveys may only note explicit physical assault without capturing the full nature of the harassment.

### Contextualizing Everyday Harassment

To better understand the normalization and everyday nature of both direct non-physical and ambient sexual harassment (witnessing), it is insightful to understand the context of these incidents—specifically, where they occurred. Witnessing sexual harassment occurred in a variety of places. The most common place this occurred was online, with students reporting witnessing 18 incidents online. Participants described 11 incidents as occurring either on campus (e.g., after class reported 4 times, in dorms reported 3 times) or off campus at a student gathering. The qualitative data further demonstrates that witnessing sexual harassment occurred in places and at times that are not commonly associated with risky places or times, such as in the library. Reporting on where jokes or comments were heard, students indicated these incidents often occurred on campus (49 times; e.g., in dorms and campus housing 5 times, at a student on-campus party 2 times, and in a professor, instructor, or teaching assistant's office 1 time). Students also heard jokes and remarks off campus. They chose the general descriptor of “off campus” 17 times. They also reported that they heard a joke or remark 2 times at a student off-campus party, and 8 times while at work (either on or off campus). Students also said they saw jokes and remarks online 20 times. Reporting on where direct, non-physical sexual harassment occurred, participants indicated it occurred on campus (10 times) and off campus (10 times), including once at a student gathering off campus. Two of these incidents occurred online or via social media.

## Discussion and Conclusion

This study represents a unique approach to the study of sexual violence on university campuses. Rather than rely on snapshots in time, whether from an incident report, a survey, or a focus group, we tracked students over the course of 60 days, opening a new window into these experiences on campus and in students’ lives. We also move beyond physical sexual violence to consider the more insidious and pervasive forms of sexual violence on campus. What we found is alarming: nonphysical and ambient sexual harassment are persistent *everyday* occurrences for women students. Sexual harassment of women students, according to our data, is not just something that happens occasionally at parties. Rather, it often happens on campus, in places like the library or the hallways outside of classrooms. These are places not readily considered to be unsafe or that are associated with risk and sexual violence. Our findings show that students not only personally experience or witness this harassment daily and that is it persistent, but taken together, their descriptions show a hostile environment where the possibility of a daily harassment is a lived, structural, and endemic reality.

Our findings also help us think what it means to be a woman university student today and what an average day might look like—the daily hassles, chronic stressors, and traumas and normalized violence women experience and witness. It occurs as a student packs up her books after class and walks into the hallway, as she studies in the library, as she takes the bus, crosses the street, or eats lunch. In other words, our findings help us think about the social context in which sexual harassment can cause stress in students’ lives occurs. This insight and our findings reinforce the need to focus on the variety of forms of sexual harassment—along a continuum of violence—and to take seriously the subtle, insidious, ambient and indirect, and normative forms of sexual harassment. Seeing harassment as an individual incident removes the data point from the realities of the student's life (on and off campus) and university context.

While this piece does not specifically link sexual harassment to mental health outcomes, our data can be contextualized alongside research show that sexual harassment (both direct and indirect, both non-physical and ambient) in university spaces is a “hazard to women's health,” having “both direct and indirect health effects, including nausea and sleeplessness, loss of self-esteem, fear and anger, feelings of helplessness and isolation, as well as nervousness and depression” (van Roosmalen & McDaniel, 1999, p. 33). Our data show that students experience this harassment daily. Women study, work, and exist in a hostile campus environment, where the possibility of experiencing or witness sexual harassment is a real and likely possibility—as demonstrated by the calendar data. Daily experiences and witnessing of sexual harassment can be a stressor in students’ lives. Such experiences “undermine women students’ economic, political, social, and personal wellbeing,” leading to “culturally and socially determined health problems” (van Roosmalen & McDaniel, 1999, p. 33).

Our data suggest that the social contexts to which policy and programs respond do not fully match the reality of the social and situational context in which harassment occurs. University responses tend to focus on most extreme forms of sexual violence, offering maxims like “don’t walk home alone,” campus walk services, and emergency blue light phones set up in places like parking lots around campus—responses that are necessary, but do not respond to non-physical sexual harassment women also frequently experience, as our data suggests. Universities in Canada are responding to a particular context in which they think sexual violence occurs, but we show that the context is different, more varied, and widespread, especially when we broaden our conceptualization of sexual violence. Typical university responses to sexual violence reflect the ways the data are commonly collected and how sexual violence and assault are conceptualized. Unfortunately, they miss the everyday, normalized sexual violence that women encounter outside the classroom, inside their instructors’ offices, and at the library; they miss the types of sexual violence that create the chilly climate women encounter on campus.

This research should be understood as part of a conversation about the university's response to sexual violence on campus. Our research shows the need for a broad conceptualization of violence against women to begin to even make sense of women's experiences on campus, and also points to the everyday nature and ambient nature of these events. These are not isolated and one-off events, they are not just individualized micro experiences of harassment. They are rather part of daily student life for women and almost to be expected. A fresh look at social context might lead universities to reconstruct responses that actually address the varying forms of sexual violence where they occur and how they occur (e.g., thinking about the escalation of non-physical harassment). Focusing on the context and varieties of experiences is one-way universities might begin to gain clarity of vision and can help move toward healthier campuses and student experiences.

In our view, broad policies that focus on reporting and prevention are not enough. Universities must create responses, training programs, and interventions that are relevant to spaces that are not normally considered risky. For example, our data show that many sexual harassment incidents occurred in places like the library or immediately after class. These situations may be surprising to students and students may require specific strategies for making sense of them and knowing how to respond. This also requires that universities conceptually shift where and what they consider spaces where students are at risk. In other words, it does not *just* occur when students are partying, consuming substances, or walking in the parking lot. Finally, mental health supports on campus must evolve to include responses and strategies to address the generalized and social stressors that students witness, hear, and are faced with every day. Because our findings reveal the insidious and normalized types of harassment, many of which may be occur in passing, students likely internalize these and carry these stressors with them in ways that are difficult to name or point to. Moreover, in order to create shifts in broader culture, we emphasize the need to bring together diverse campus stakeholders (from students to administrators) and to focus on environments (not just individuals) as Hirsch et al. (2021) do in their SPACE (Sexual Prevention and Community Equity) Toolkit. This innovative and new approach uses a public health approach and is rooted in equity and addresses the role of power in assault. This toolkit focuses on special regulations, residential spaces, social spaces, public spaces, and the digital spaces that make up students’ lives.

When thinking about the implications of this study, multiple considerations should be acknowledged. Although generalization was not the aim of this study, it is worth noting that the data are from a convenience sample and, as such, may not represent the experiences of the larger study body or students at other universities. The fluctuation of participation over the course of the study could also impact the generalizability of the findings (Newcomb et al., 2018), but such fluctuation does not affect the validity of the findings in most cases (Ohly et al., 2010). Additionally, it is possible that persons extremely interested in the topic would have been more likely to participate in this study, especially given the time commitment involved. Swim et al. (2001) speculate that those with an interest in the research topic at hand may “self-select into situations in which they are less likely to encounter gender prejudice” (p. 42). As such, it is possible that an interest in sexual harassment could have reduced the likelihood of experiencing sexual harassment because the women in the study may have “self-selected” out of situations where harassment may be more likely to occur. Alternatively, it is possible that an interest in harassment may have heightened participants’ awareness of harassment making them more likely to recognize its occurrence. However, since we asked questions about specific events (e.g., “did you hear someone make a sexual remark or tell a sexual joke that you found insulting or offensive?” “have you been sexual harassed (this includes *unwelcome sexual advances, requests for sexual favors, and other verbal conduct of a sexual nature*)?” emphasis added), the participants’ responses *should* reflect what occurred rather than prior interest, interpretations, preconceptions, or understandings of harassment. As such, any interest in the topic should not undermine the validity of the findings. It is, however, still possible that preconceptions about sexual harassment may have influenced individual responses.

Future research should also consider the role peers and other students can have in interventions. For example, researchers might investigate the role that senior students can have in changing campus culture and modeling intervention actions in everyday common spaces on campus. Researchers might conduct an extensive diary study that covers the course of the entire academic year with a larger group of students. Such a study could identify additional situational risks associated with the academic schedule—for example, orientation and frosh week, exam periods, and so forth. Expanding the number of students participating would also provide a more accurate assessment of the pervasiveness and the nature of sexual harassment. Future research would also benefit from distinguishing between multiple experiences occurring on the same day versus a single experience encompassing multiple forms of contact. The diary tool this study uses can also be applied to research on unwanted sexual experiences occurring in other settings, such as the workplace. As this study demonstrates, this tool can capture the subtler and everyday forms of sexual violence that are not always easily recalled after a significant amount of time has passed. Such research would help identify the situational factors that contribute to these experiences occurring in other settings among non-students. Furthermore, this tool could be used to assess other negative experiences, such as experiences of everyday racism and other forms of discrimination.

## Notes

1. Including “when a victim is unable to consent (e.g., due to age, illness) or refuse (e.g., due to physical violence or threats)” or incapacitation (Basile et al., 2014, p. 1).

2. We define sexual assault as “Intentional touching, either directly or through the clothing, of the genitalia, anus, groin, breast, inner thigh, or buttocks of any person without his or her consent, or of a person who is unable to consent or refuse” (Basile et al., 2014, p. 12).

3. Personal communication from an anonymous reviewer for earlier version of this manuscript. This review/reviewer was not from *Violence Against Women*.
